# Targeted alleviation of ischemic stroke reperfusion via atorvastatin-ferritin Gd-layered double hydroxide

**DOI:** 10.1016/j.bioactmat.2022.05.012

**Published:** 2022-05-25

**Authors:** Li Wang, Baorui Zhang, Xueting Yang, Shuaitian Guo, Geoffrey I.N. Waterhouse, Guangrong Song, Shanyue Guan, Aihua Liu, Liang Cheng, Shuyun Zhou

**Affiliations:** a Key Laboratory of Photochemical Conversion and Optoelectronic Materials, Technical Institute of Physics and Chemistry, Chinese Academy of Sciences, Beijing, 100190, China; b Beijing Neurosurgical Institute and Beijing Tiantan Hospital, Capital Medical University, China National Clinical Research Center for Neurological Diseases, Beijing, 100070, China; c Institute of Functional Nano & Soft Materials (FUNSOM), Jiangsu Key Laboratory for Carbon-Based Functional Materials & Devices, Soochow University, Suzhou, 215123, China; d School of Chemical Sciences, The University of Auckland, Auckland, 1142, New Zealand

**Keywords:** Layered double hydroxide, ROS scavenger, Atorvastatin, Ischemic stroke, Multimodal imaging

## Abstract

In acute ischemic stroke therapy, potent neuroprotective agents are needed that prevent neural injuries caused by reactive oxygen species (ROS) during ischemic reperfusion. Herein, a novel 2D neuroprotective agent (AFGd-LDH) is reported, comprising Gd-containing layered double hydroxide nanosheets (Gd-LDH, as a drug nanocarrier/MRI contrast agent), atorvastatin (ATO, as a neuroprotective drug) and the ferritin heavy subunit (FTH, as a blood brain barrier transport agent). Experiments revealed AFGd-LDH to possess outstanding antioxidant activity, neuroprotective properties, blood‒brain barrier transit properties, and biocompatibility. *In vitro* studies demonstrated the ROS scavenging efficiency of AFGd‒LDH to be ∼90%, surpassing CeO_2_ (50%, a ROS scavenger) and edaravone (52%, a clinical neuroprotective drug). Ischemia‒reperfusion model studies in mice showed AFGd‒LDH could dramatically decrease apoptosis induced by reperfusion, reducing the infarct area by 67% and lowering the neurological deficit score from 3.2 to 0.9. AFGd-LDH also offered outstanding MRI performance, thus enabling simultaneous imaging and ischemia reperfusion therapy.

## Highlights

• The simple stepwise method was used to construct AFGd-LDH by the confinement of atorvastatin and the ferritin heavy subunit (FTH) with Gd-LDH.
• AFGd-LDH demonstrated outstanding antioxidant activity and ROS scavenging efficiency.
• AFGd-LDH offered neuroprotective properties to dramatically decrease apoptosis induced by reperfusion.
• AFGd-LDH presented blood‒brain barrier transit properties and outstanding MRI performance, thus enabling simultaneous imaging and ischemia reperfusion therapy.

## Graphical abstract

**Figure:**
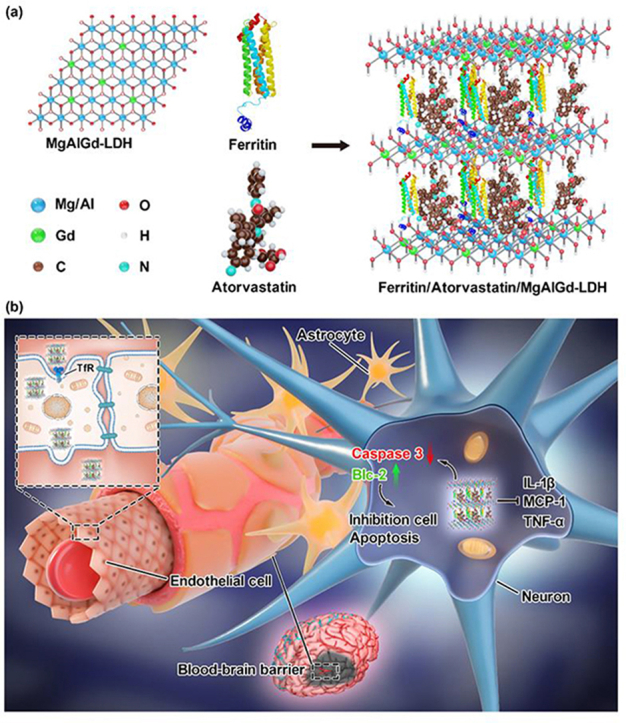

## 1 Introduction

Globally, one in six people will have a stroke in their lifetime, with more than 13.7 million new strokes and 5.8 million stroke-related deaths occurring each year [1]. Ischemic strokes, accounting for approximately 80% of all strokes [2], cause both disability and death, with the care of ischemic stroke victims becoming a huge burden on modern societies due to rapid population growth coupled with aging populations [[3], [4], [5]]. The best method for ischemic stroke treatment is early vascular recanalization through thrombolysis and (or) thrombectomy to restore cerebral blood flow and protect brain tissue and function [[6], [7], [8], [9]]. However, only about 21.5% of acute stroke patients arrive at the emergency department within 3 h of a stroke, thus intravenous thrombolysis rates remain low (only about 2.4% within 3 h) [10]. The treatment window is limited to 4.5 h after a stroke for intravenous thrombolysis and 6 h after a stroke for thrombectomy [[11], [12], [13], [14]], since the efficacy of these treatments is restricted by secondary injuries mainly caused by oxidative stress (OS) and an inflammatory reaction [15]. Therefore, stroke patients have a limited treatment window with an immediate intervention needed to limit further neurological deterioration.

After the restoration of blood supply to brain tissues (reperfusion) following an ischemic stroke, tissue damage can occur (often referred to as an ischemia-reperfusion injury (IRI) or reoxygenation injury). This is attributed to excessive generation of reactive oxygen species (ROS), such as superoxide anion radicals (•O_2_^−^), hydroxyl radicals (•OH), hydrogen peroxide (H_2_O_2_), and nitric oxide (NO), in the ischemic penumbra, leading to increased neuronal death through protein oxidation, DNA damage, and lipid peroxidation [[16], [17], [18]]. These ROS create a severe oxidative storm in the body, which cannot be eliminated without intervention. If these ROS can be captured after vascular recanalization, the prognosis after an ischemic stroke will be improved. Certain nanomaterials, due to their small size, and ROS scavenging functionality, care capable of capturing ROS *in vivo*, thereby reducing their potential to cause damage. Nanomaterials with different morphologies and compositions have been fabricated for this purpose, including 9-aminoacridine/L-PEG-cRGD [19], carbon dots (CDs) [20], heparin nanoparticles with immobilized VEGF clusters [21], cerium dioxide (CeO_2_) [22], and a neuroprotective agent NR_2_B_9_C modified with red blood cells [23]. Despite some progress, many of these systems suffer from low ROS capture efficacy and low biocompatibility. Further, because of blood-brain barrier (BBB) (an innate barrier between blood plasma and neuron cells formed by the walls of brain microvascular endothelial cells (BMECs), pericyte, and astrocyte end-foot processes [24].), drug therapies developed to date for ischemic stroke therapy have been largely limited to small lipophilic molecules that can passively diffuse across endothelial cell membranes [[25], [26], [27]]. To allow exploitation of nanotechnology in the prevention of ischemic stroke‒reperfusion injuries, composite nanomaterials need to be discovered that can not only transit the BBB with high ROS capture efficiency, but also allow *in vivo* visualization.

Layered double hydroxides (LDHs) are inorganic layered materials, with nominal formula M^2+^_1−x_M^3+^_x_ (OH)_2_ (A^n−^_x/n_)·mH_2_O. The LDH construction is composed of positively charged brucite‒like layers [28], with the structure able to accommodate a diverse range of metal cations and interlayer anions. This allows LDHs with different electronic structures and specific surface properties to be synthesized. Functionalization of LDHs with functional drug molecules or other moieties provides a robust multi-functional platform for *in vivo* imaging and therapy, including ROS capture [[29], [30], [31], [32], [33], [34], [35]]. Due to their 2D structure, LDH sheets can serve as nanocarriers, offering great potential for the treatment of cerebral ischemic stroke. Atorvastatin, a drug known for its lipid lowering capabilities and thus reduction of cardiovascular disease, has demonstrated neuroprotective effects following ischemic stroke [[36], [37], [38]]. The ferritin heavy chain (FTH), a natural iron storage protein complex, binds to the transferrin receptor 1 (TfR1) of endothelial cells to transverse the BBB via endocytosis [39,40]. Inspired by the excellent properties of these materials, we hypothesized that functionalization of LDH nanosheets with the ferritin heavy subunit and Atorvastatin should yield an organic‒inorganic hybrid (AFGd-LDH) with excellent performance for the prevention of ischemia-reperfusion injury caused by ROS. Further, by incorporating Gd^3+^ cations in the LDH nanosheets, simultaneous MRI imaging and ischemia reperfusion therapy should be possible.

**Scheme 1:**
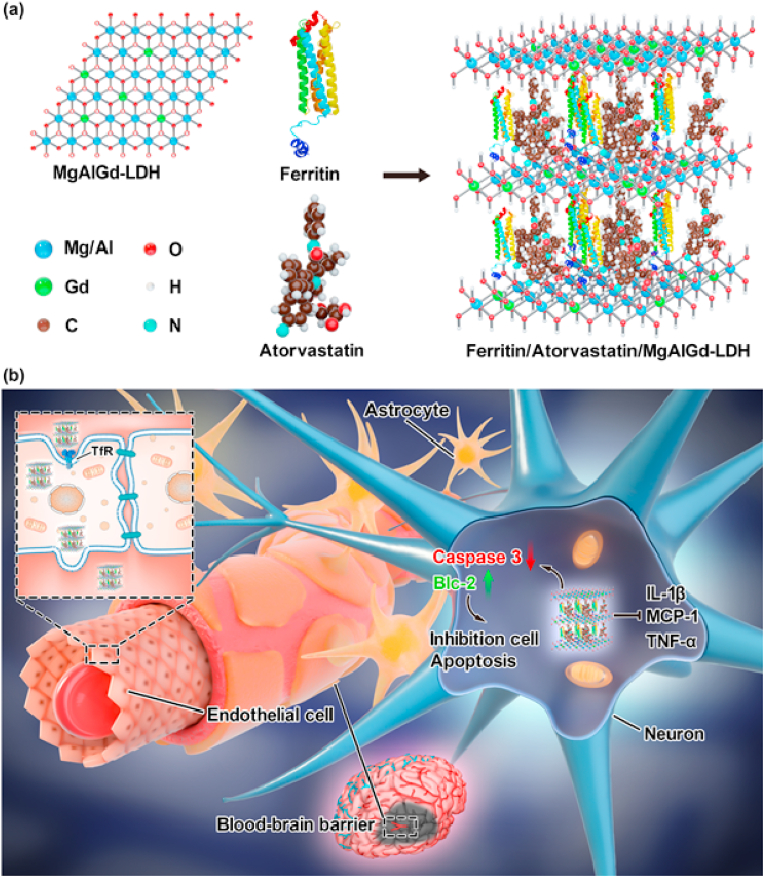
Schematic illustration of the preparation of Ferritin/Atorvastatin/MgAlGd-LDH (denoted as AFGd‒LDH) and its neuroprotective function mechanisms for treatment reperfusion-generated damage after ischemic stroke.

Herein, we report the proof-of-concept development of such a multi-functional imaging and therapeutic agent. The stepwise synthesis of AFGd-LDH is illustrated in Scheme 1. To begin, we prepared a MgAlGd-LDH via a simple one-step method (in which the Gd^3+^ cations occupied some of the M^3+^ sites in the LDH structure). For simplicity, we denote MgAlGd-LDH herein simply as Gd-LDH. Surface modification of Gd-LDH with Atorvastatin (ATO, an anti-inflammatory and anti‒lipid peroxidation drug) and the ferritin heavy subunit (FTH) yielded AFGd-LDH, which demonstrated outstanding performance as an MRI imaging agent and as a therapeutic for alleviating brain reperfusion injury. As hypothesized, the rationally designed AFGd-LDH nanocomposite possessed excellent ROS scavenging efficiency, superior to both CeO_2_ (an effective ROS scavenger) and edaravone (a clinical neuroprotective drug). In addition, the abundant hydroxyl groups on the surface of AFGd-LDH imparted the hybrid with high biocompatibility and stability. Both *in vitro* and *in vivo* experiments verified that AFGd-LDH was able to reduce neuron apoptosis and the oxidative damage in the cortex of mice brains, thus offering a viable and safe neuroprotective therapy for the effective suppression of ischemic stroke‒reperfusion injury. In addition, AFGd-LDH could effectively reduce the inflammation response during ischemia‒reperfusion injury. As a further demonstration of its versatility, AFGd-LDH offered outstanding MRI performance. Together, this study not only introduces a novel approach for simultaneous MRI imaging and ischemia reperfusion therapy, but also sheds light on neuroprotective mechanisms that can be exploited to avoid reperfusion injury after ischemic stroke.

## 2 Results and discussion

**Fig. 1:**
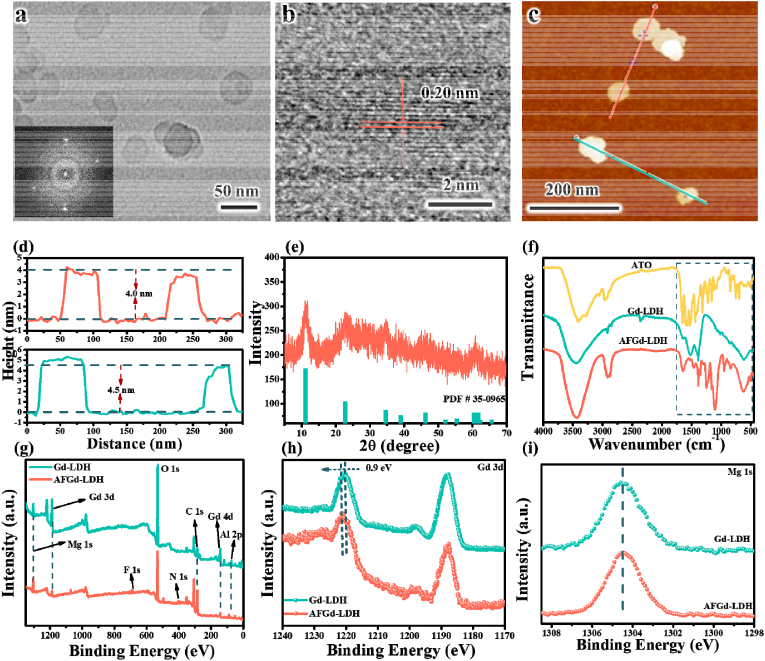
**Structure, morphology and chemical composition of AFGd-LDH.** (a–b) TEM and HRTEM images of AFGd-LDH. (c) AFM image and (d) the corresponding height profiles of AFGd-LDH. (e) XRD pattern of AFGd-LDH. (f) FTIR spectra for ATO, Gd-LDH and AFGd-LDH. (g) XPS survey spectra for Gd-LDH and AFGd-LDH. (h) Gd 3 d and (j) Mg 1s XPS spectra for Gd-LDH and AFGd-LDH.

A wide variety of techniques were applied to follow the stepwise synthesis of Gd-LDH and the AFGd-LDH nanohybrid. Transmission electron microscopy (TEM) showed Gd-LDH and AFGd-LDH to consist of ultrathin hexagonal nanosheets (Figs. 1a and S1), with lateral dimensions of around 40 nm and 50 nm, respectively. These lateral dimensions were in excellent accord with particle sizes determined by the dynamic light scattering (DLS) (Fig. S2). High-resolution TEM (HRTEM) revealed lattice spacings of ∼0.20 nm (Fig. 1b), corresponding to LDH (018) planes. Atomic force microscopy (AFM) was used to determine the thickness of the Gd-LDH and AFGd-LDH nanosheets. The average thickness of the Gd-LDH and AFGd-LDH nanosheets were ∼2.0 nm and ∼4.0 nm, respectively. This increased thickness of AFGd-LDH can be attributed to the insertion of drug molecules into the LDH interlayer regions (Fig. 1c and d and S3). Results confirm the successful synthesis of AFGd-LDH hybrid via incorporation of ATO and ferritin between/on the ultrathin Gd-LDH nanosheets. The X-ray diffraction (XRD) pattern for AFGd-LDH showed a characteristic set of peaks at 2*θ* = 11.34, 22.8, 34.7, 39.1, 46.4, and 60.6° (Fig. 1e), which could readily be assigned to the (003), (006), (012), (015), (018), and (110) planes of LDH. It was worth noting that the full width at half maximum (FWHM) of the peak at *θ* = 22.8° widened after functionalization with ATO and FTH (Fig. S4), confirming the successful synthesis of AFGd-LDH hybrid via surface modification of the LDH basal surfaces. The loading efficiency of ATO in AFGd-LDH was determined to be about 9 wt% by the UV–Vis absorption spectroscopy detection of atorvastatin at 240 nm (Fig. S5). Zeta potential measurements were next performed on dispersions of Gd-LDH and AFGd-LDH at neutral pH (pH 7) to examine their respective surface charges. All samples were charged at neutral pH, sufficient to be stable against aggregation (net charge >30 mV) (Fig. S6). The zeta potentials of Gd-LDH and AFGd-LDH were determined to be +46.3 mV and +41 mV, respectively. This result can be attributed to the immobilization of a negatively charged drug (−50.2 mV) onto positive charged Gd-LDH nanosheets (Fig. S6), indicating interaction between Gd-LDH and atorvastatin/ferritin heavy subunit was mainly electrostatic attraction. Scanning transmission electron microscopy (STEM)‒EDS mapping of AFGd-LDH (Fig. S4) indicated that Mg, Al, Gd, O, and C were uniformly distributed in the synthesized nanocomposite.

Next, Fourier‒transform infrared (FTIR) spectroscopy was applied to identify the functional groups present on the surface of AFGd‒LDH, information which was essential for understanding its excellent dispersion properties in aqueous media. After functionalization of Gd-LDH with ATO and FTH, several characteristic peaks associated with ATO and FTH appeared in the FTIR spectra. The most prominent amongst there were amide‒related modes in the 1650-1200 cm^−1^ region (both ATO and FTH contain amide groups), as well as the modes due to the –COOH group and aromatic rings of ATO (Fig. 1f). The ATO peaks at 1700 cm^−1^ to 500 cm^−1^ red-shifted on binding to Gd-LDH, suggesting possible intercalation of ATO into the interlayer of Gd-LDH. Further, comparing the spectra of ATO with AFGd-LDH, the peaks at 1518 cm^−1^ (C–N stretching) and 1430 cm^−1^ (O–H bending) for AFGd-LDH were red-shifted (Fig. S5) [41], suggesting that ATO likely formed hydrogen bonds with Gd-LDH. In addition, AFGd-LDH showed an intense and broad feature centered around 3440 cm^−1^ due to O–H stretching modes (Fig. 1f), which contributed to the excellent dispersibility and stability of AFGd-LDH in aqueous solution. The dispersibility and stability of AFGd-LDH was further confirmed by Zeta potential measurements over 1 week. As shown in Fig. S9, AFGd-LDH shows very good stability against aggregation, remaining well-dispersed after 7 days without stirring.

To further probe the surface composition of the AFGd-LDH nanohybrid, X-ray photoelectron spectroscopy (XPS) analysis was carried out. The survey spectra (Fig. 1g) of Gd-LDH and AFGd-LDH showed the presence of Mg, Al, Gd, O, and N, typical for the Gd-LDH sample (actually a MgAlGd-LDH) with nitrate ions in the interlayers. For AFGd-LDH, extra carbon was present along with fluorine, the latter being present in ATO (Fig. 1g). The Fe in FTH was masked by the surrounding protein. The XPS signals of Gd‒LDH were attenuated after surface functionalization with ATO and FTH, consistent with XPS being a surface analytical technique probing the top few nanometers of samples. Core-level XPS spectra were collected over the Gd 3 d, Mg 1s, and Al 2p regions allowed examination of the valence states of the metal cations in the Gd-LDH and AFGd-LDH samples. The Gd 3 d, Mg 1s, and Al 2p spectra for Gd-LDH and AFGd-LDH (Fig. 1h–j and S10) had similar peak shapes and binding energies, indicating the presence of Gd^3+^, Mg^2+^, and Al^3+^ in both samples. It could be concluded that the functionalization with ATO and FTH did not change the valence states of the metals in the Gd-LDH support, again consistent with the nanohybrid system utilizing electrostatic and hydrogen-bonding interactions between components.

**Fig. 2:**
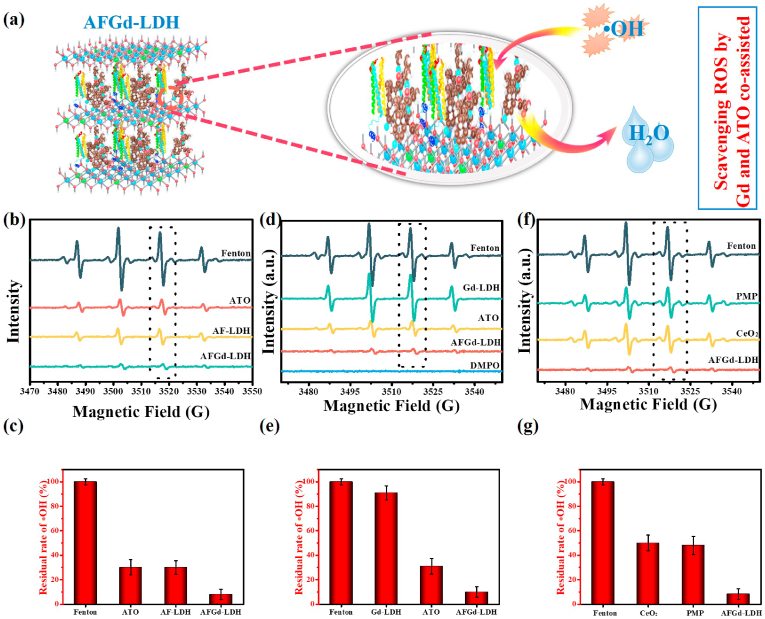
**ROS scavenging activity of AFGd-LDH.** (a) ROS scavenging mechanism of AFGd-LDH. (b) EPR spectra for DMPO-OH adducts formed in the presence of ATO, ATO/LDH (without Gd), and AFGd-LDH, respectively (all samples tested at the same ATO concentration of 100 μg/mL). •OH radicals were produced via the Fenton reaction using a Fe^2+^/H_2_O_2_ method and discovered with DMPO. (c) ROS scavenging efficiency determined from ESR results in (b). (d) EPR spectra for DMPO-OH adducts formed in the presence of Gd-LDH, ATO and AFGd-LDH, respectively. (e) ROS scavenging efficiency determined from ESR results in (d). (f) EPR spectra for DMPO-OH adducts formed in the presence of edaravone (PMP), CeO_2_ and AFGd-LDH, respectively (all samples tested at the same concentration 500 μg/mL). (g) ROS scavenging efficiency determined from ESR results in (f). (Error bar, n = 3).

Since we aimed to develop a treatment to prevent ischemia-reperfusion injury, the ROS scavenging capabilities of samples demanded detailed investigation. An •OH scavenging assay was used, with electron paramagnetic resonance (EPR) spectroscopy used for quantification. In the assay, •OH radicals were generated by the Fe^2+^/H_2_O_2_ system and quantified using DMPO (5,5′-dimethylpyrroline1-xide) as the trap. In the absence of a radical scavenger, the Fenton reaction generated strong EPR signals for the DMPO-OH adduct (Fig. 2b-g). The intensity of the EPR signal for DMPO-OH adduct was greatly reduced when ATO was included in the Fe^2+^/H_2_O_2_ system, consistent with the known ROS scavenging ability of ATO. For AFGd-LDH, the EPR signal was very weak, implying that the assembled nanohybrid possessed excellent ROS (•OH) scavenging properties. Interactions between ATO and Gd cations in the Gd-LDH nanosheets might have contributed to enhanced ROS scavenging activity of AFGd-LDH. To explore this possibility, an ATO/LDH composite was prepared as a control sample (using a MgAl-LDH without Gd^3+^). The ATO/LDH nanohybrid possessed •OH scavenging properties similar to ATO (Fig. 2b and c). Gd-LDH offered negligible ROS (•OH) scavenging ability (Fig. 2d and e). The high ROS scavenging efficiency of AFGd-LDH thus mainly resulted from the presence of –NH, –OH and active ortho- and para-sites on the aromatic rings for ATO, which can react with ROS and deactivate ROS [42]. Whilst the mechanism for the enhanced ROS scavenging by AFGd-LDH is unclear, it likely involves some form of direct interaction between Gd cations in the LDH nanosheets and ATO. The uniform dispersion ATO over the Gd-LDH nanosheets likely contributed the excellent scavenging performance of AFGd-LDH, by maximizing sites for •OH neutralization (Fig. 2a). To further probe the ROS scavenging performance of AFGd-LDH, further experiments were conducted using CeO_2_ (an effective and recyclable ROS scavenger) and edaravone (a clinical neuroprotection drug) (Fig. 2f and g). The ROS scavenging performance of AFGd-LDH was ∼90%, superior to both CeO_2_ (50%) and edaravone (52%), highlighting the huge potential of the nanohybrid in the prevention of reperfusion induced injury in ischemic stroke patients.

**Fig. 3:**
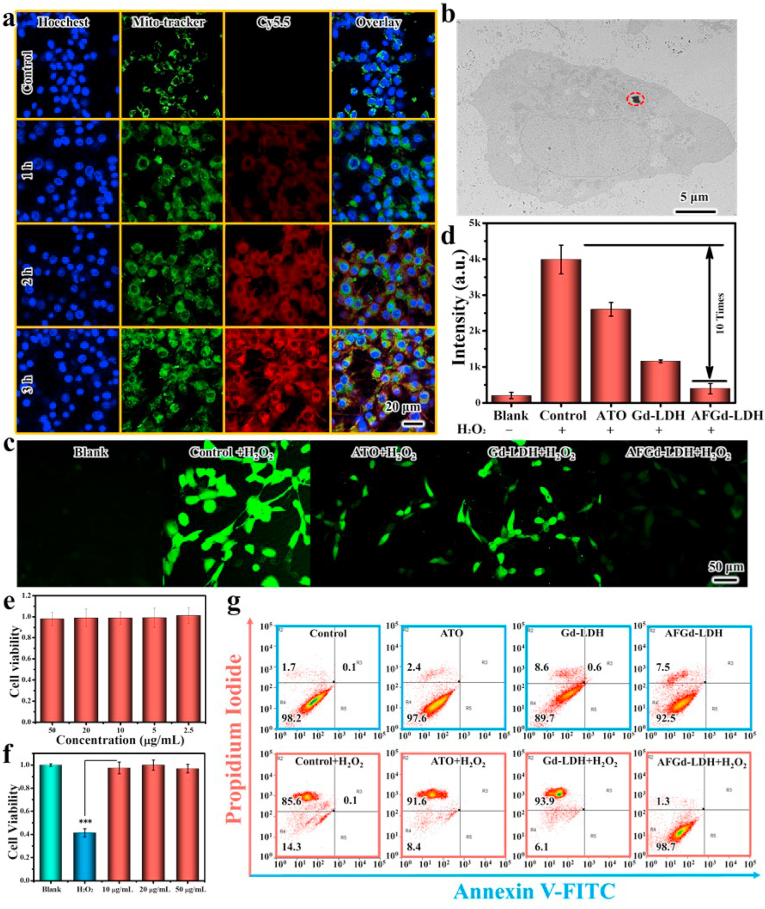
**Endocytosis and ROS scavenging effects of AFGd-LDH *in vitro*.** (a) Intracellular trafficking of Cy5.5-labeled AFGd-LDH in PC12 cells. (b) TEM image of AFGd-LDH internalized in PC12 cells. (c–d) ROS images inside PC12 cells after treatment with different samples (20 μg/mL) after 6 h incubation. (e) The cell viability of PC12 cells incubated with AFGd-LDH (2.5, 5, 10, 20, 50 μg/mL) for 48 h. (f) The cell viability of PC12 cells incubated with H_2_O_2_ and various concentrations (10, 20, 50 μg/mL) of AFGd-LDH for 48 h. (g) FITC/PI staining to assess the improving of H_2_O_2_- caused apoptosis by AFGd-LDH (20 μg/mL). PC12 cells were treated with H_2_O_2_, ATO + H_2_O_2_, Gd-LDH + H_2_O_2_ and AFGd-LDH + H_2_O_2_ for 48 h.

Inspired by the excellent ROS scavenging properties of AFGd-LDH, we next examined the *in vitro* performance of the nanohybrid. In neurophysiology, PC12 cells with neuroendocrine cells features are typically utilized. Thus, PC12 cells were selected as a cell model for ischemic stroke injury. TEM (Fig. 3b) showed that AFGd-LDH could be internalized by PC12 cells, entering mitochondria and accumulating in lysosomes. To further explore the internalization of AFGd-LDH, we prepared Cy5.5-labeled AFGd-LDH to allow confocal imaging. Mitotracker (green fluorescence) and Hoechst (blue fluorescence) were selected to stain PC12 cells. As shown in Fig. 3a, no red fluorescence from Cy 5.5 was detected for the control group, whilst weak red fluorescence was observed after 1 h incubation of Cy5.5-labeled AFGd-LDH with PC12 cells. The green fluorescence signal of the mitotracker overlayed the red fluorescence signal of Cy 5.5 after the 1 h incubation period. As the incubation time was increased from 1 h to 3 h, the red fluorescence intensified indicating internalization of AFGd-LDH via mitochondria‒mediated endocytosis. The cytoxicity of AFGd‒LDH was next explored, by incubating the nanohybrid with PC12 cells for 48 h (Fig. 3e). The PC12 cell viability was above 90% even at a high concentration of 50 μg/mL, confirming that AFGd-LDH possessed good biocompatibility. To investigate the *in vitro* antioxidant ability of the nanocomposites (particularly important for ischemic stroke injury therapy), an *in vitro* ROS oxidation-mediated PC12 cell damage model was used. Results confirmed the strong protective effect of AFGd-LDH. As illustrated in Fig. 3f, H_2_O_2_ (15 μM) inhibited PC12 cell proliferation, resulting in cell viability of only 41.3%. AFGd-LDH protected PC12 cells from H_2_O_2_‒induced oxidative damage even at a low AFGd-LDH concentration of 10 μg/mL. Furthermore, we used flow cytometry to analyze possible reversal of H_2_O_2_‒induced apoptosis by AFGd-LDH. No obvious cell apoptosis was detected after incubating cells with ATO, Gd-LDH, or AFGd-LDH (Fig. 3g). After adding H_2_O_2_, strong cell apoptosis was observed for cells incubated with ATO + H_2_O_2_ and Gd-LDH + H_2_O_2_. In contrast, the cell apoptosis was only 1.3% for cells treated with AFGd-LDH + H_2_O_2_. Accordingly, AFGd-LDH could protect PC12 cells from oxidative damage due to its efficient free radical scavenging abilities. Next, we scrutinized the ability of AFGd-LDH to capture intracellular ROS using 2′,7′-dichlorodihydrofluorescein-diacetate (DCFH-DA). The presence of H_2_O_2_ enhanced the ROS signal in PC12 cells, with no green fluorescence signal observed in the control group (Fig. 3c and d). Both the ATO + H_2_O_2_ and Gd-LDH + H_2_O_2_ groups showed negligible ROS scavenging ability. In contrast, the cells treated with AFGd-LDH showed very weak green fluorescence, further evidence of the strong free radical scavenging abilities of the synthesized nanocomposite.

To scrutinize the biocompatibility of AFGd-LDH *in vivo*, the hemocompatibility was evaluated by a hemolytic activity assay under physiological conditions. The hemoglobin release was almost negligible when blood samples were treated with AFGd-LDH at different concentrations (Fig. S11). Compared with the recognized safe value (5%), the hemolytic activity relatively lower (∼3%) even at a high AFGd-LDH concentration (100 ppm). Results confirm that AFGd-LDH possessed superb hemocompatibility and was able to be used as an intravenous injection (i.v.) drug for *in vivo* investigates. For the mice *in vivo* studies, we intravenouslyadministered AFGd-LDH and performed blood panel analysis and biochemical indicators of the liver functions. The blood panel analysis results (Fig. S12A) showed that the amount of white blood cells, red blood cells, platelets, neutrophils, lymphocytes, and the hemoglobin content were not statistically different from the control group. In addition, administration of AFGd-LDH caused no change in the levels of alanine transaminase (ALT), aspartate transaminase (AST), triglyceride (TG), gamma-glutamyl transpeptidase (GGT), high-density lipoprotein cholesterol (HDL-C), or low-density lipoprotein cholesterol (LDL-C) (Fig. S12B). These indicators suggest that AFGd-LDH did not cause acute liver injury to mice after blood exposure. These data further verified that AFGd-LDH was *in vivo* biocompatible.

**Fig. 4:**
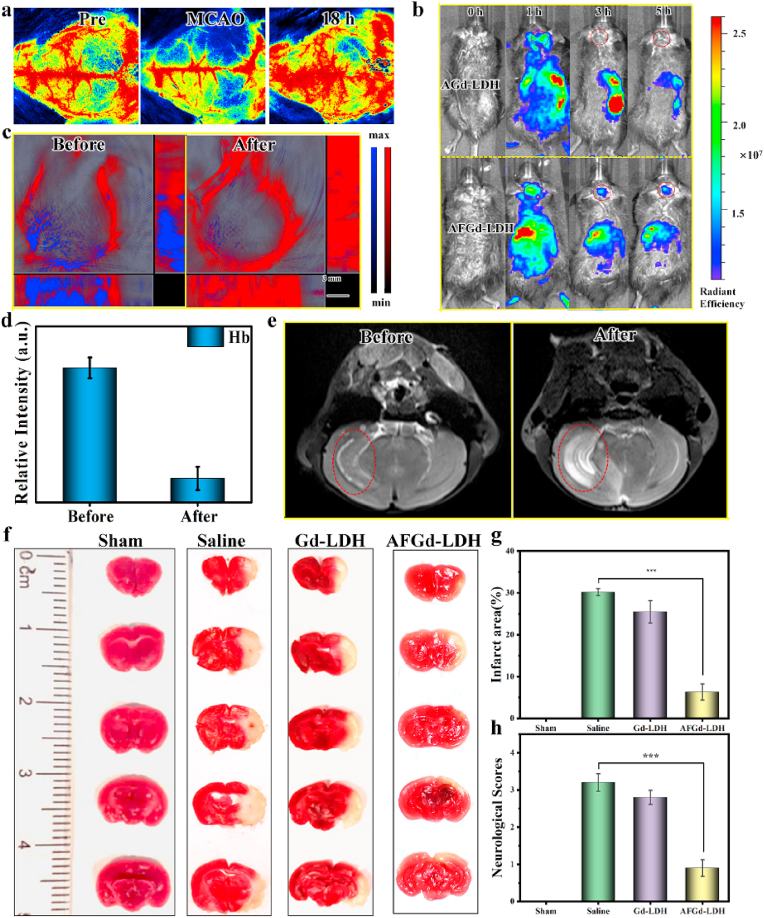
**AFGd-LDH reduces ischemic stroke infarct areas through decreasing ROS-caused oxidative damage**. (a) Cerebral blood flow (CBF) measured by laser speckle imaging in a tMCAO mice model before and after restored perfusion for 18 h. (b) In vivo imaging of Gd-LDH and AFGd-LDH, respectively. (c–d) Photoacoustic imaging of tMCAO mice before and after the injection of AFGd-LDH. (e) MRI imaging of tMCAO mice before and after the injection of AFGd-LDH. (f) Photographs of brain slices by TTC-stained after cure with sample (n = 3). (g) Resultant infarct zones of various treatment determined by imaging (n = 3). (h) Neurological deficit scores of tMCAO mice by different treatments at 3 days (n = 3). *p < 0.05, **p < 0.01 and ***p < 0.001.

To confirm the brain protection properties of AFGd-LDH *in vivo*, ischemia-reperfusion after stroke was simulated by a transient middle cerebral artery occlusion (tMCAO) mice model. Briefly, the tMCAO mice model was established through inserting a filament into the carotid bifurcation along the internal carotid artery to the origin of the middle cerebral artery for 90 min, followed by the removal of the filament to restore blood flow to the middle cerebral artery territory. To visually observe the process of ischemia-reperfusion, we measured the cerebral blood flow (CBF) by laser speckle imaging. The cortical CBF value returned to preoperative level 18 h after reperfusion, confirming the successfully established tMCAO mice model (Figs. 4a and S13). The ferritin heavy subunit in AFGd-LDH, allowed targeting of the transferrin 1 (TfR1) receptor at the BBB, thereby ensuring accumulation of AFGd-LDH in the brain of tMCAO mice. To verify that AFGd-LDH could penetrate the BBB, we investigated the *in vivo* biodistribution of AFGd-LDH using the tMCAO mice model. The photosensitizer indocyanine green (ICG) with long-wavelength emission properties was selected as the label for both AGd-LDH and AFGd-LDH. As shown in Fig. 4b and Fig. S14, both the AGd-LDH and AFGd-LDH reached the brain site 1 h after intravenous (*i.v*) injection, explained by the transient BBB opening after cerebral ischemia reperfusion. However, no fluorescence signal was found at the brain site after 3 h for the AGd-LDH group, indicating that AGd-LDH failed to penetrate the BBB. In addition, the free ICG group produced no fluorescence signal at the brain site after intravenous (*i.v*) injection (Fig. S14a), indicating that AFGd-LDH and ICG need to be linked together during blood circulation to successfully penetrate the BBB. A strong fluorescence signal was detected at the brain site for the AFGd-LDH group after 3 h and up to 5 h. All these findings suggested that AFGd-LDH could accumulate in the brain by targeting the BBB through the ferritin heavy subunit. To further illustrate the ability of AFGd-LDH to alleviate anoxic conditions, we used photoacoustic imaging (PAI) to monitor brain oxygen levels in tMCAO mice. A clear blue fluorescence signal was monitored in the tMCAO mice brains in the absence of any treatment, indicating severe hypoxia (Fig. 4c and d). After the administration of AFGd-LDH, the intensity of blue fluorescence was significantly decreased with strong red fluorescence being observed, indicating that AFGd-LDH could alleviate the hypoxia environment, making the nanohybrid functional for alleviating brain ischemia-reperfusion injury. Imaging-guided treatment of ischemic stroke is highly desirable, in that it allows both treatment of the condition and also visualization of brain activity. Due to the high-spin of the Gd^3+^ cations in the LDH layers, direct coordination of water to Gd^3+^ cations should result in long longitudinal relaxation times. Therefore, AFGd-LDH was expected to serve as a useful MRI contrast agent to allow monitoring of the brain in real time. To examine the MRI property of AFGd-LDH, *in vivo* MR imaging of the tMCAO mice 3 h after surgery was performed. As shown in Fig. 4e, the signal of the infarction lesion increased sharply after *i. v* injection of AFGd‒LDH, indicating that AFGd-LDH was an effective MRI contrast agent for imaging the brain.

To further confirm the neuroprotective effect of AFG-LDH, we again used the tMCAO mice model to mimic ischemia-reperfusion after a stroke. The neurological deficit scores and infarct areas of tMCAO mice were explored at 3 days after the AFGd-LDH treatment. The neurological deficit scores were determined based on the Zea-Longa criteria. As shown in Fig. 4f, the 2,3,5-triphenyltetrazolium chloride (TTC) staining verified that tMCAO mice injected with saline showed a large-area infarction, whilst the brain infarct area after AFGd-LDH treatment was significantly decreased. Through quantitative analysis, the infarct area in the saline group was ∼42.3%, while the infarct area in the AFG-LDH group was only ∼15.7% (Fig. 4g), i.e. around one-third that of the saline group. The neurological deficit scores of tMCAO mice after AFGd-LDH treatment also decreased significantly (from 3.2 to 0.9, Fig. 4h).

Malondialdehyde (MDA) is the final product of lipid peroxidation in living organisms, affecting the mitochondrial respiratory chain complex and key enzyme activities in mitochondria [43]. As a result, we further scrutinized the production of MDA in the infarct cortex of the tMCAO mice 3 days after the AFGd-LDH treatment. It could be found that the MDA content of the AFGd-LDH group was much lower than in the saline group and the Gd-LDH group (Fig. S15). Glutathione peroxidase (GSH) and superoxide dismutase (SOD) are both essential antioxidant enzymes in living organisms, playing key roles in balancing oxidative and antioxidant systems and reducing free radical damage [44]. The SOD and GSH contents in the infarct cortex of tMCAO mice at 3 days after the AFG-LDH treatment were considerably upper than in the saline group (Fig. S16 and Fig. S17). All the above results showed that AFGd-LDH increased the antioxidant capacity in the infarcted area and cleared excessive free radicals responsible for ischemia-reperfusion injury after stroke.

**Fig. 5:**
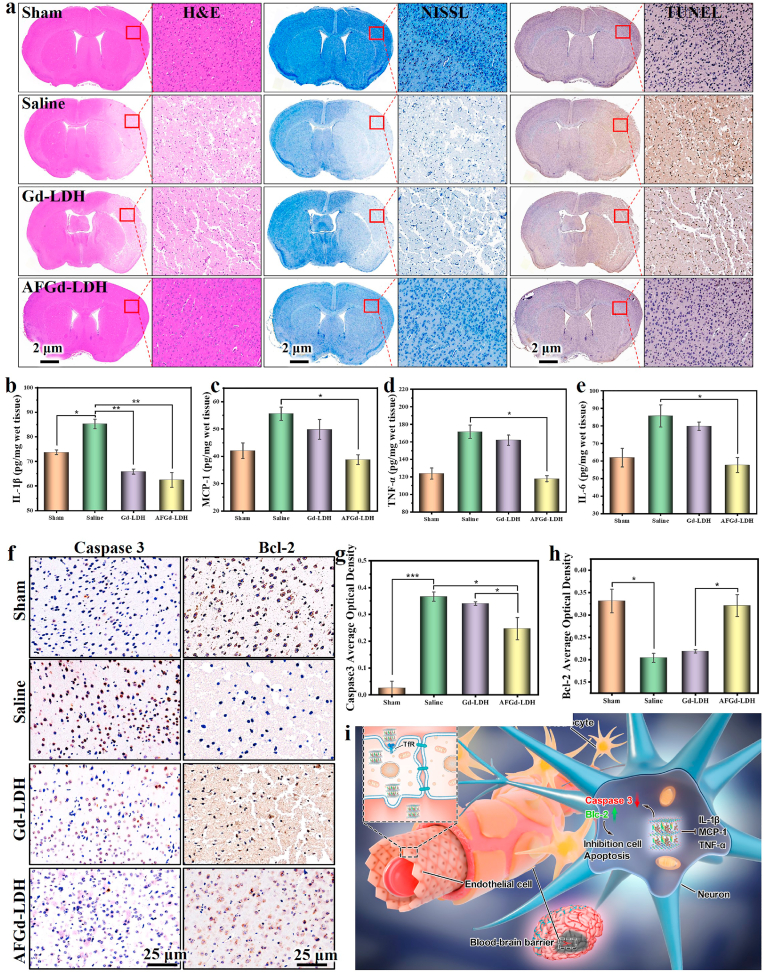
**Therapeutic effects of AFGd-LDH and its inhibition on inflammation response caused through reperfusion in ischemic stroke.** (a) H&E, Nissl and TUNEL staining of brain tissues from various groups. Inflammatory cytokines of IL-1β (b), MCP-1 (c), TNF-α (d),IL-6 (e) in the infarct part of different treatment groups (n = 3). *p < 0.05, **p < 0.01 and ***p < 0.001. (f–h) The expression level of caspase 3 and Bcl-2 of tMCAO mice from various groups investigated by immunohistochemical staining. (i) Schematic illustration for neuroprotective application mechanisms against reperfusion‒induced injury in ischemic stroke of AFGd-LDH.

In addition, the neuroprotective effect of AFGd-LDH was further investigated through pathological analysis. Firstly, hematoxylin and eosin (H&E) staining of the infarcted cortex in tMCAO mice treated with saline or Gd-LDH displayed heavy necrosis at the infarction area. In contrast, the AFGd-LDH group showed a much smaller area of necrosis (Fig. 5a). The neuron damage was also investigated via Nissl staining in the infarcted area of tMCAO mice. The number of intact neurons in the infarcted area of tMCAO mice treated with saline or Gd-LDH was low compared to the sham-operated group, with the intact neurons showing a disordered arrangement (Fig. 5a). However, after treatment with AFGd-LDH, the neuron damage in the brain cortex was minimal. From the TUNEL (terminal deoxynucleotidyl transferase-mediated deoxyuridine triphosphate nick-end labeling) staining, the infarcted cortex area of tMCAO mice in the saline group demonstrated apoptotic features, whereas the AFGd-LDH group showed remarkably reduced apoptosis of neurons (Fig. 5a).

In order to examine whether AFGd-LDH could decrease the apoptosis induced by ischemic stroke reperfusion, Bcl-2 and Caspase 3 levels and were examined by immunohistochemical staining 3 days after AFGd-LDH treatment. Relative to the Gd-LDH group and saline group, the expression levels of Bcl-2 in the AFGd-LDH group were much higher and comparable to the sham-operated group (Fig. 5f). The Caspase 3 expression levels in the AFGd-LDH group were significantly lower than in the saline group. Through quantitative analysis, the average optical densities (AOD) of Bcl-2 increased and Caspase 3 decreased after treatment with AFGd-LDH (Fig. 5g and h). This result indicated that AFGd-LDH was beneficial for reducing neuronal cell apoptosis during ischemia-reperfusion injury. Next, we aimed to examine whether AFGd-LDH alters inflammatory activation caused by the reperfusion in ischemic stroke 3 days after treatment. Therefore, we scrutinized the expression levels of inflammatory cytokines in the infarct cortex, including interleukin 1β (IL-1β), monocyte chemoattractant protein-1 (MCP-1), tumor necrosis factor α (TNF-α), and interleukin 6 (IL-6). It was found that AFGd-LDH reduced the levels of IL-1β, MCP-1, TNF-α, and IL-6 in the infarct cortex of tMCAO mice (Fig. 5b-e). Hence, AFGd-LDH could effectively reduce the inflammation response during ischemia‒reperfusion injury. Meanwhile, the main organs of the saline, Gd-LDH, and AFGd-LDH treatment groups were gathered for staining and histopathological analysis (Fig. S18). There was no significant histological difference among these groups. Together, our findings demonstrate that AFGd-LDH offered a safe and effective neuroprotective effect against ischemia-reperfusion injury after stroke *in vivo*.

## 3 Conclusion

In summary, a AFG-LDH nanocomposite with excellent ROS scavenging abilities was successfully synthesized by functionalizing Gd-containing LDH nanosheets with atorvastatin and the BBB targeting protein Ferritin heavy subunit. Interaction between Gd cations in the LDH and ATO (likely involving hydrogen bonding), and the uniform dispersion ATO over the Gd-LDH support, enhanced the ROS (•10.13039/100002264OH) scavenging properties of the nanocomposite. AFG-LDH demonstrated excellent performance for reducing neurons apoptosis and oxidative damage in the brain cortex (tMACO mice model) under the guidance of magnetic resonance imaging. This work introduces a novel approach for the fabrication of MRI‒guided nano therapeutics with potential neuroprotective effects, highlighted herein by the application of AFGd-LDH for the suppression/treatment of the ischemia-reperfusion injury.

## 4 Experimental section

*Synthesis of Gd-LDH.* MgAlGd-LDH (denoted herein simply as Gd-LDH) was synthesized according to a previously reported method. Briefly, 40 mL of a mixed aqueous solution containing Mg(NO_3_)_2_·6H_2_O (0.03 M), Al(NO_3_)_3_·9H_2_O (0.01 M) and Gd(NO_3_)_3_·6H_2_O (0.2 g) was added to 40 mL of an aqueous 25 vol% formamide solution containing NaNO_3_ (0.4 g) under constant stirring at room temperature. Then, 35 mL of 0.25 M NaOH was added to the metal salt solution with stirring at 80 °C. After reaction for 6 h, the MgAlGd-LDH (i.e Gd-LDH) product was collected by centrifugation at 10,000 r/min and then washed 3 times with water and then ethanol. Finally, the Gd-LDH product was redispersed in water to give a dispersion with a concentration of 2 mg/mL.

*Synthesis of AGd-LDH:* A solution of atorvastatin (ATO) in water was added to a Gd-LDH dispersion (1 mg/mL) under magnetic stirring at room temperature for 24 h. The AGd-LDH product was collected by centrifugation and re-dispersed in water at a concentration of 1 mg/mL for further use.
*Synthesis of AFGd-LDH*: Ferritin (100 μL, 1 mg/mL) was added to a dispersion of AFGd-LDH in water (1 mg/mL, 5 mL). After stirring for 24 h in the dark, the AFGd-LDH product was collected by centrifugation (10,000 rpm, 10 min) and washed repeatedly with DI water.
*ICG-labelling of AFGd-LDH*: *ICG* (0.1 mg) was added to a dispersion of AFGd-LDH in water (100 μg/mL, 5 mL). After stirring for 24 h in the dark, the AFGd-LDH/ICG product was collected by centrifugation (10,000 rpm, 10 min) and washed repeatedly with DI water.

### 4.1 *In vitro* experiments

The *in vitro* cytotoxicity of AFGd-LDH was assessed on PC12 cell lines. After seeding into a 96-well plate by pipetting, the PC12 cells were exposed to series doses of AFGd-LDH for 48 h. After further incubation for 48 h, the CCK-8 solution was added to each well. The cell viability was then calculated as the ratio of the absorbance of the wells versus the absorbance of the control. Furthermore, the CCK-8 assay was used to examine the effect of AFGd-LDH against H_2_O_2_-induced cytotoxicity in PC12 cells. Briefly, After cell adherence, diverse concentrations of AFGd-LDH and H_2_O_2_ (15 μM) were incubated with PC12 cell for 48 h, and cell viability was determined by CCK-8 assay.

*Evaluation of intracellular ROS*: Briefly, PC12 cells were seeded in glass bottom cell culture dish and incubated for 24 h. The cells were pretreated with H_2_O_2_ (15 μM) for 12 h, and then incubated with different samples for 6 h and next stained with DCFH-DA for 30 min. The blank control group did not have drug, with all other conditions similar to the experimental group. The cells were examined for ROS generation via confocal imaging.

### 4.2 *In vivo* experiments

All the animal experiments were approved by the Experimental Animal Ethics Committee of Beijing Neurosurgical Institute and performed under legal protocols. The transient middle cerebral artery occlusion (tMCAO) mice model was established to assess the protective effects for AFGd-LDH treatment ischemia-reperfusion after stroke. The tMCAO mice model was implemented by inserting a filament with a silicone tip into the carotid bifurcation along the internal carotid artery to the origin of the middle cerebral artery for 90 min, after which the filament was removed to restore blood flow to the middle cerebral artery territory. The sham operation group wasset up, uniform with the usage of the tMCAO mice model barring the silicone filament embolization. The tMCAO mice were split into three groups: saline, Gd-LDH, and AFGd-LDH. All samples dispersed injected at the caudal vein after reperfusion.

## CRediT authorship contribution statement

**Li Wang:** designed the experiments, synthesized the materials and performed the performance experiments, performed the cells experiments, wrote and revised the manuscript; all authors reviewed and edited the manuscript. **Baorui Zhang:** designed the experiments, performed animal experiments; all authors analyzed and interpreted the data. **Xueting Yang:** performed the cells experiments. **Shuaitian Guo:** performed the cells experiments. **Guangrong Song:** performed animal experiments; all authors analyzed and interpreted the data. **Shanyue Guan:** oversaw all research, designed the experiments, wrote and revised the manuscript; all authors reviewed and edited the manuscript. **Aihua Liu:** oversaw all research. **Liang Cheng:** oversaw all research, designed the experiments, wrote and revised the manuscript; all authors reviewed and edited the manuscript. **Shuyun Zhou:** wrote and revised the manuscript; all authors reviewed and edited the manuscript.

## Declaration of competing interest

The authors declare no competing financial interest.
