# Supplementary material for: Targeted alleviation of ischemic stroke reperfusion via atorvastatin-ferritin Gd-layered double hydroxide
Source: Bioact Mater. 2022 May 25;20:126–36. doi: 10.1016/j.bioactmat.2022.05.012 (PMC9136047; doi:10.1016/j.bioactmat.2022.05.012)
Supplement: Multimedia component 1 [file mmc1.docx]

**Supporting Information**

**Targeted alleviation of Ischemic Stroke reperfusion via Atorvastatin-Ferritin Gd-Layered Double Hydroxide**

Li Wang^1,3†^, Baorui Zhang^2†^, Xueting Yang^1^, Shuaitian Guo^1^, Geoffrey I.N. Waterhouse^4^, Guangrong Song^2^, Shanyue Guan^1^*, Aihua Liu^2^*, Liang Cheng^3^,* and Shuyun Zhou^1^

^1^Key Laboratory of Photochemical Conversion and Optoelectronic Materials, Technical Institute of Physics and Chemistry, Chinese Academy of Sciences, Beijing, 100190, China.

^2^Beijing Neurosurgical Institute and Beijing Tiantan Hospital, Capital Medical University, China National Clinical Research Center for Neurological Diseases, Beijing 100070, China.

^3^Institute of Functional Nano & Soft Materials (FUNSOM), Jiangsu Key Laboratory for Carbon-Based Functional Materials & Devices, Soochow University, Suzhou 215123, China

^4^School of Chemical Sciences, The University of Auckland, Auckland 1142, New Zealand.

*Correspondence authors: [guanshanyue@mail.ipc.ac.cn](mailto:guanshanyue@mail.ipc.ac.cn), [liuaihuadoctor@163.com](mailto:liuaihuadoctor@163.com), [lcheng2@suda.edu.cn](mailto:lcheng2@suda.edu.cn)

**^†^** These authors contributed equally to this work.

**Experimental Section**

Reagents and Chemicals

Mg(NO_3_)_2_·6H_2_O, Al(NO_3_)_3_·9H_2_O, Gd(NO_3_)_3_·6H_2_O, NaNO_3_, NaOH, Cyanine 5.5 (Cy5.5), 2,7-dichlorodi-hydrofluorescein diacetate (DCFH-DA) and Hoechest stain were purchased from Sigma-Aldrich Corporation. RPMI medium1064 basic (RPMI-1064), fetal bovine serum (FBS), and phosphate buffer solution (PBS) were obtained from Beijing Solarbio Science and Technology Co., Ltd. H-ferritin protein was purchased from MerryBio Co., Ltd. Cell Counting Kit (CCK-8), Mitobright LT green was purchased from Dojindo China Co., Ltd. Deionized (DI) water was used in all experiments.

*In vitro* experiments.

The *in vitro* cytotoxicity of AFGd-LDH was assessed on PC12 cell lines. PC12 cells were incubated in RPMI 1640 medium with FBS (10%), penicillin (100 U/mL) and streptomycin (50 U/mL) at 37 °C in a 5% CO_2_ incubator. Cells were first incubated in a 25 cm^2^ cell-culture flask and then subsequently seeded into a 96-well plate (1×10^4^ cells/well) by pipetting. After seeding, the PC12 cells were exposed to series doses of AFGd-LDH for 48 h. After further incubation for 48 h, a mixture of CCK-8 and RPMI-1064 (1:10) was added to each well. The cell viability was then calculated as the ratio of the absorbance of the wells versus the absorbance of the control. Absorbances at 450 nm were measured using a multi-mode microplate reader (Thermo Multiskan FC). The cytotoxicity was calculated by averaging six individual reads in six identical wells. Furthermore, the CCK-8 assay was used to examine the effect of AFGd-LDH against H_2_O_2_-induced cytotoxicity in PC12 cells. Briefly, PC12 cells (1 × 10^4^ per well) were seeded in 96-well plates. After cell adherence, various concentrations of drugs and H_2_O_2_ (15 μM) were successively added to each well and incubated for another 48 h, and cell viability was determined by CCK-8 assay.

*Loading efficiency study of ATO on AFGd-LDH:* The supernatant from the drug loading experiments and product washings were all collected for measurement of the drug loading efficiency by the Ultraviolet-Visible (UV-Vis) absorption spectroscopy technique. Firstly, standard ATO solutions (30, 25, 20, 15, 10 μg/mL) was converted into a UV-absorbing compound (240 nm) through treatment with a dilute hydrochloric acid solution. Subsequently, a standard absorption curve of ATO was obtained. Similarly, the absorption at 240 nm of the supernatant obtained after reaction with AFGd-LDH and product washing was measured. ATO drug loading efficiency according the following equation:

(M_initial drug_ ̶ M_drug in supernatant_)/M_initial drug_ ×100%

*In vivo* experiments

Neurological scores of tMCAO mice after treatment with AFGd-LDH were determined using Zea-Longa criteria at 3 days. Subsequently, brain tissues were removed and washed with saline three times, and then brain tissue was cut into five 2-mm slices. The brain slices were transferred into a 2% 2,3,5-triphenyltetrazolium chloride (TTC) solution in PBS and incubated at 37 °C for 20 min. After TTC staining, the brain slices were fixed in a 4% paraformaldehyde solution for imaging and quantification analyses. Sections of the brain tissues underwent hematoxylin and eosin (H&E) staining, Nissl staining, and terminal deoxynucleotidyl transferase dUTP nick end labeling (TUNEL) staining. The expression levels of Caspase 3 and Bcl-2 in the brain infarcted cortex of tMCAO mice were examined by immunohistochemistry (IHC) staining.

The contents of MDA, SOD, and GSH in the brain tissue of the tMCAO mice were examined using the ELISA kits. Briefly, the brain homogenate in the infarct sites was prepared using a tissue homogenizer, then centrifuged at 12,000 rpm for 10 min to obtain the supernatant. The protein concentration in the supernatant was then determined using the bicinchoninic acid assay. Then, the brain homogenates with the same protein concentration in different-treatment MCAO mice were examined to detect the contents of malondialdehyde (MDA), superoxide dismutase (SOD), and reduced glutathione (GSH) using the ELISA kits according to the manufacturers specified methods. Finally, the concentrations of inflammatory factors including Interleukin-1β (IL-1β), monocyte chemoattractant protein-1 (MCP-1), Tumor necrosis factor α (TNF-α), and Interleukin (IL-6) in brain homogenates were examined using ELISA kits according to the manufacturers specified methods.

Characterization

The morphology of the AFGd-LDH composite was examined using transmission electron microscopy (TEM, JEOL JEM-2100F operated at 150 kV). Powder X-ray diffraction (XRD) patterns were collected on a Bruker X-ray diffractometer (D8 focus, Cu Kα, *λ* = 0.15178 nm) with a step of 0.1^o^ s^−1^. XPS analyses were carried out on an ESCALAB 250Xi (Thermo Scientific), equipped with an Al Kα X-ray source. Confocal fluorescence images were obtained on a Nikon A1R Eclipse Ti confocal laser scanning microscope fitted with a 40× water immersible objective. A Thermo Multiskan FC was used to investigate cell viability. Photoacoustic imaging was performed on a multispectral optoacoustic tomographic (MSOT) real-time imaging system (inVision 128, Germany).

Statistical Analysis

Statistical significance was assessed using a one-way ANOVA analysis on SPSS 16.0 software. The difference was statistically significant if the probability value was less than 0.05 (*p* < 0.05). Mean values and standard deviations (SD) were calculated for triplicate experiments. All data are presented as mean ± SD.

**Supporting Figures**

**
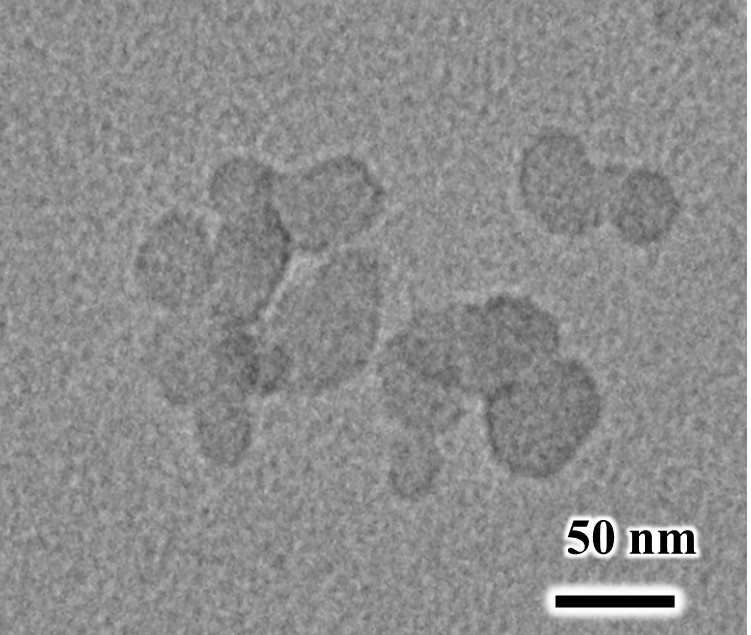
**

**Figure S1**.TEM image of Gd-LDH.


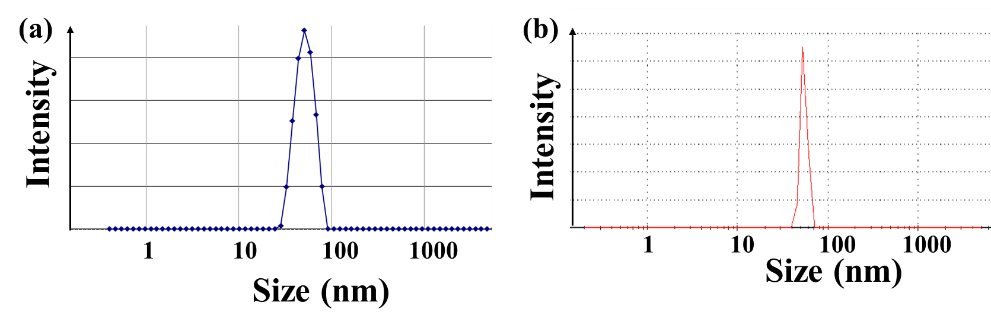


**Figure S2**. Particle size distribution of (a) Gd-LDH and (b) AFGd-LDH determined by dynamic light scattering (DLS).


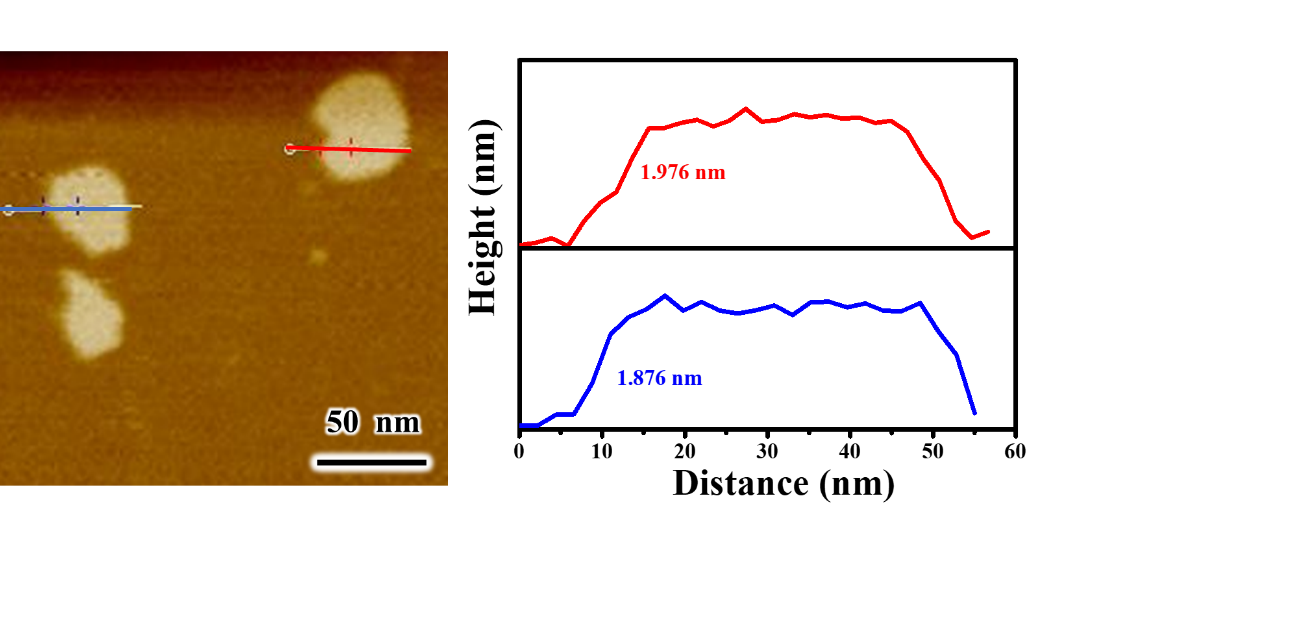


**Figure S3.** AFM image and the corresponding height profiles of Gd-LDH.


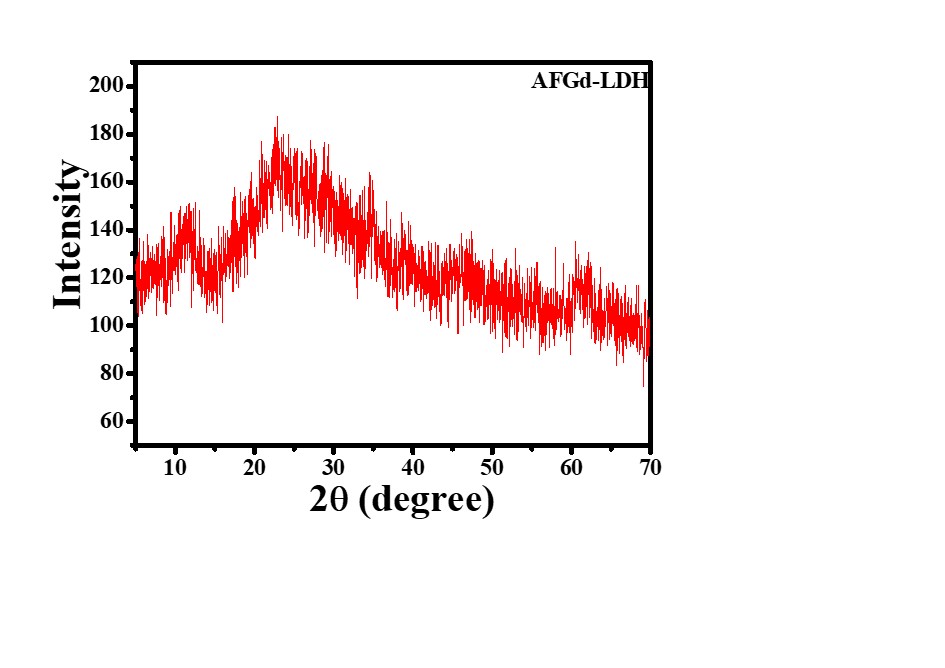


**Figure S4**. Powder XRD pattern for AFGd-LDH.


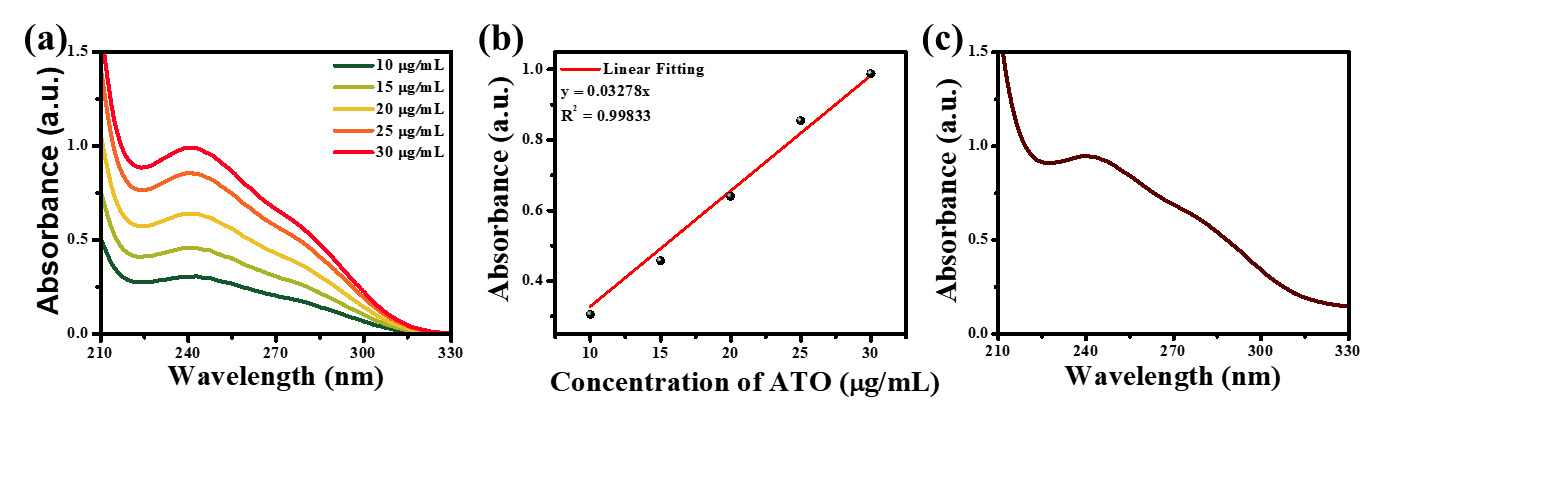


**Figure S5.** (a) UV-Vis spectra of ATO solutions with different concentration following reaction with dilute hydrochloric acid. (b) Corresponding standard absorption curve of ATO. (c) UV-Vis absorption spectrum of AFGd-LDH.


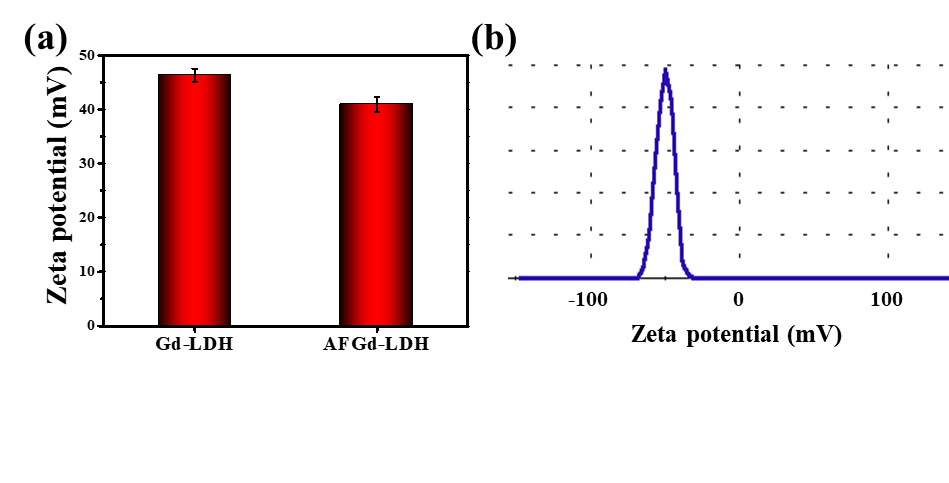


**Figure S6**. (a) Zeta potentials for Gd-LDH and AFGd-LDH at pH=7 (Error bars represent mean ± SD, n = 6 per group). (b) Zeta potential for ATO solution.


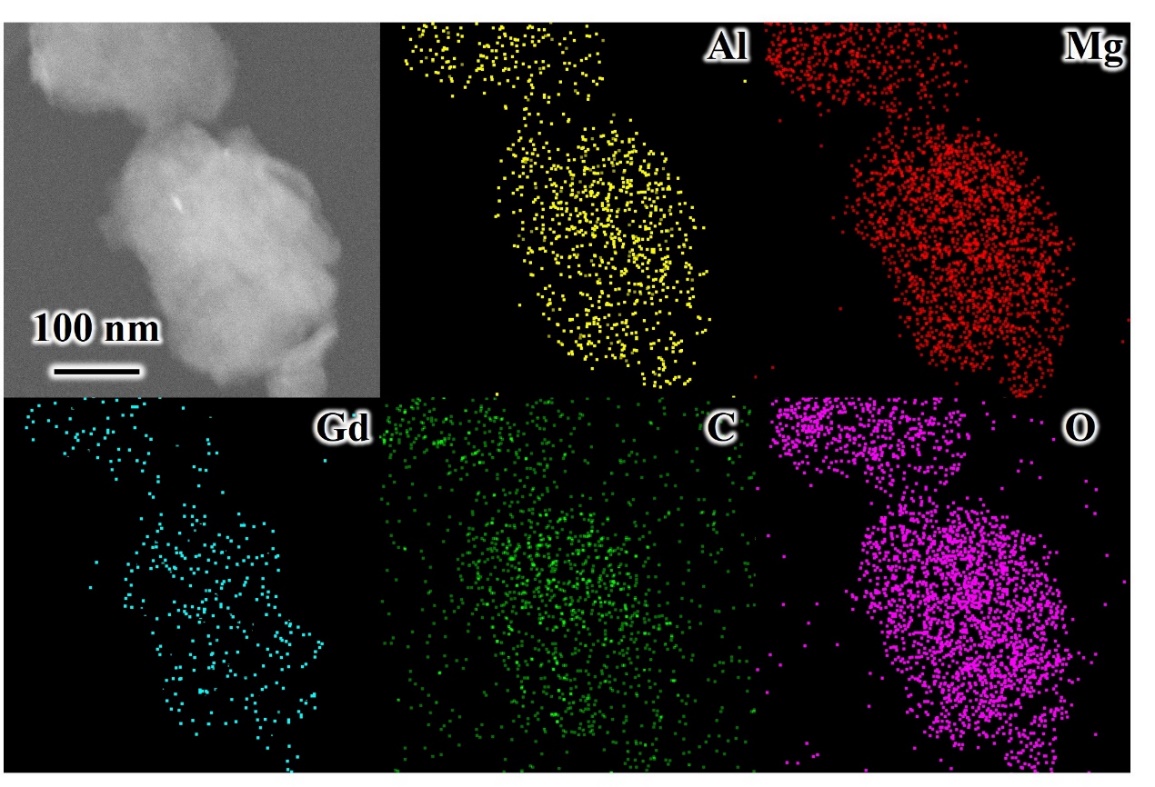


**Figure S7**. STEM-EDS element mapping of AFGd-LDH.


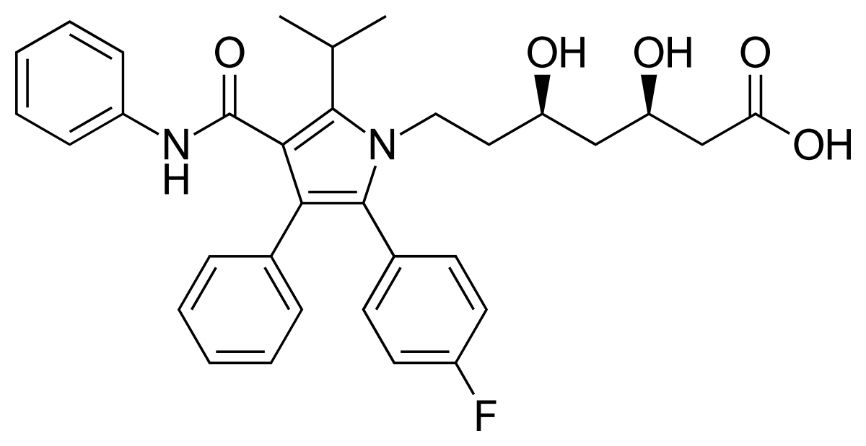


**Figure S8**. Structure of atorvastatin (top) and FTIR spectra of ATO and AFGd-LDH (bottom).





**Figure S9**. Zeta potential of AFGd-LDH solution over 7 days.


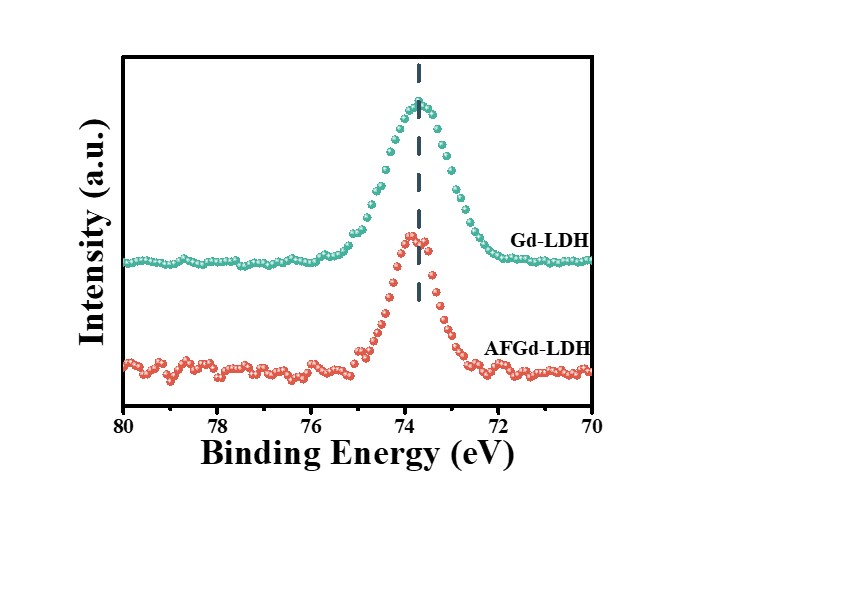


**Figure S10**. Al 2p XPS spectra for Gd-LDH and AFGd-LDH.


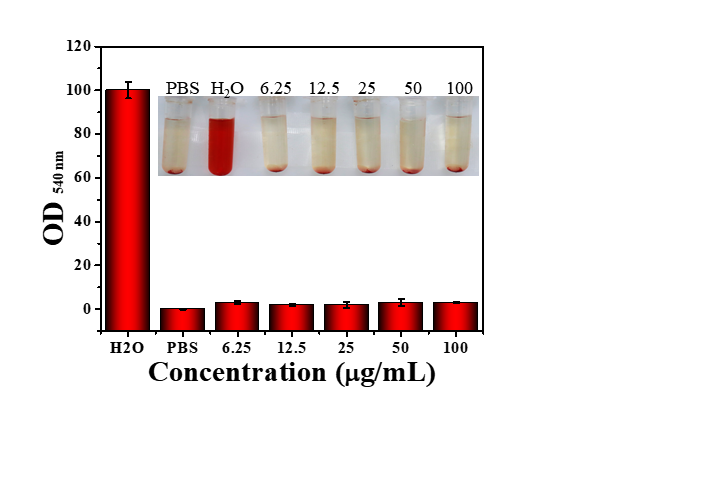


**Figure S11**. Hemolysis activity of AFGd-LDH.


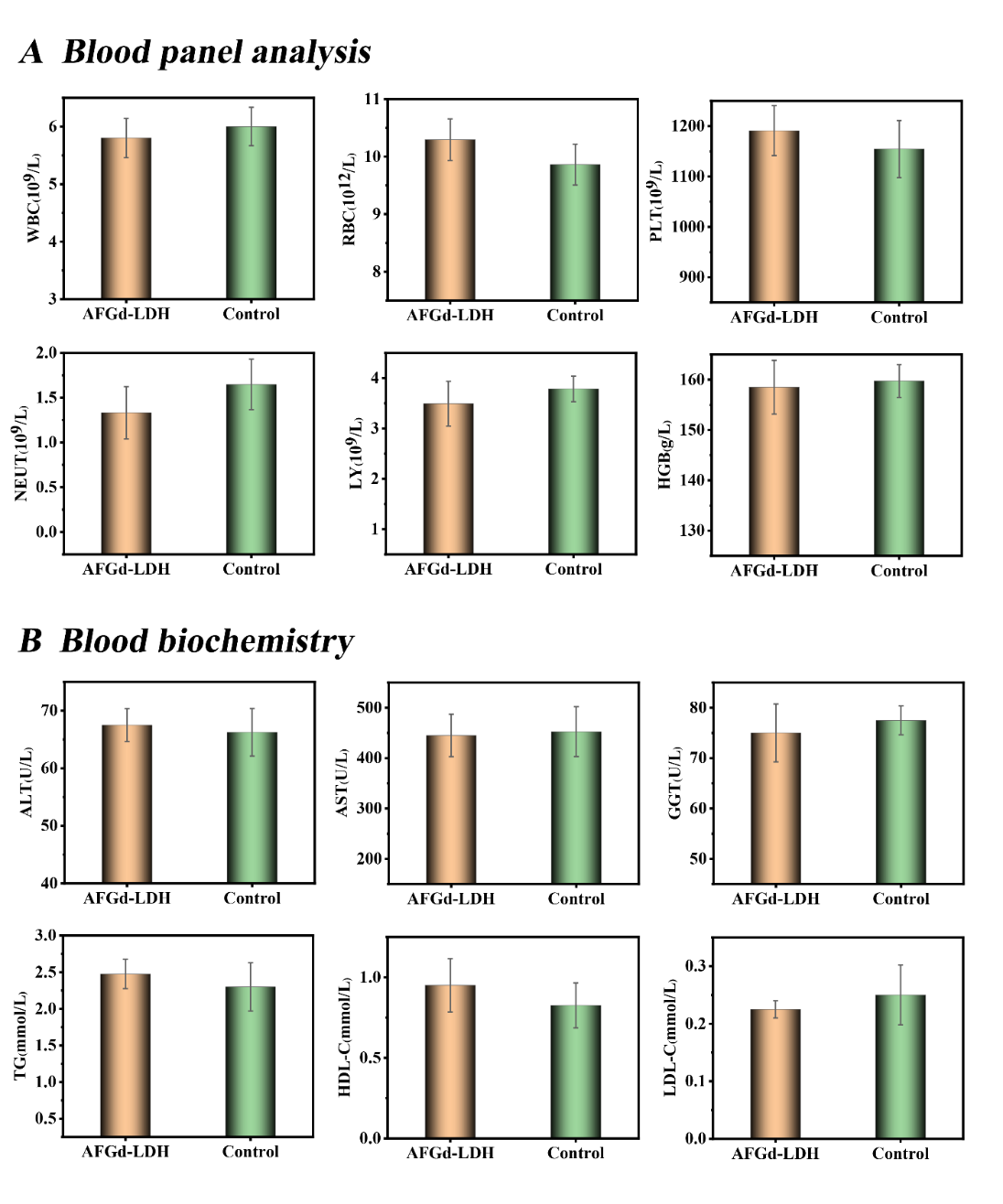


**Figure S12**. Blood panel analysis (**A**) and biochemical indicators (**B**) of the liver functions post 3 h i.v. injection of AFGd-LDH. ALT, alanine transaminase; AST, aspartate transaminase; TG, triglyceride; GGT, gamma-glutamyl transpeptidase; HDL-C, high-density lipoprotein cholesterol; LDL-C, low-density lipoprotein cholesterol; WBC, [white](javascript:;) [blood](javascript:;) [cell](javascript:;); RBC, red blood cell; PLT, platelets; NEU, neutrophil; LY, lymphocyte; and HGB, hemoglobin.


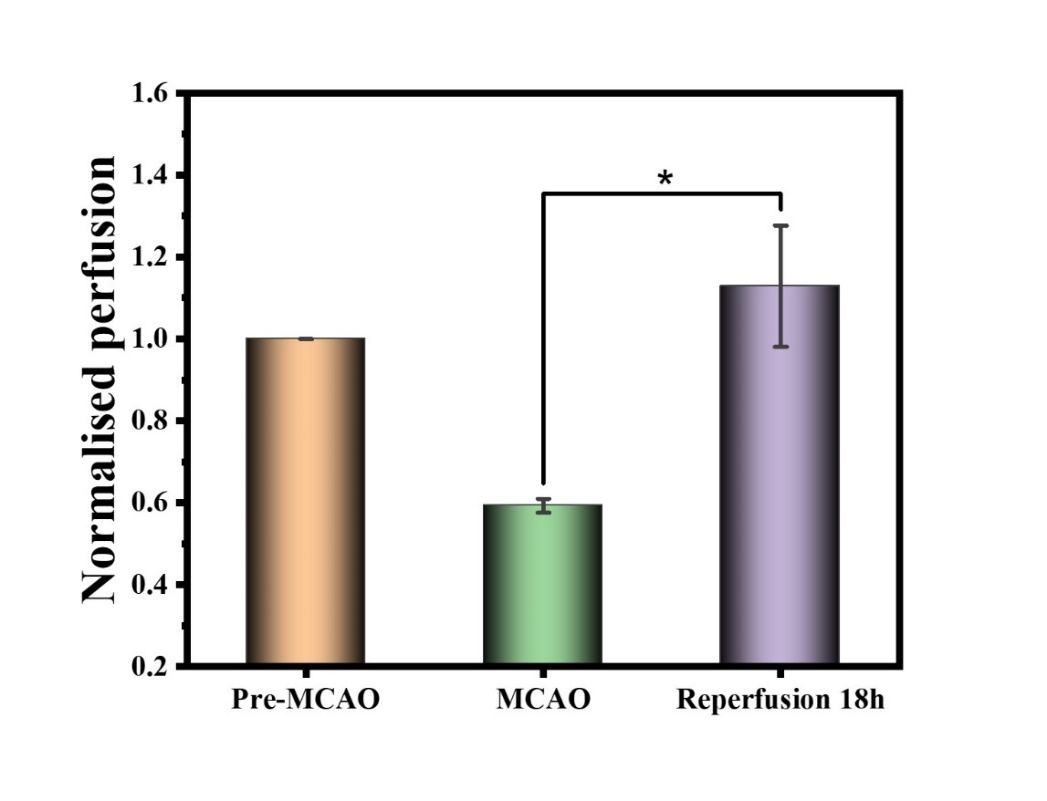


**Figure S13**. Summary showing Pre-MCAO, MCAO, reperfusion for 18 h CBF as percentage of the pre-ischemic baseline.

**
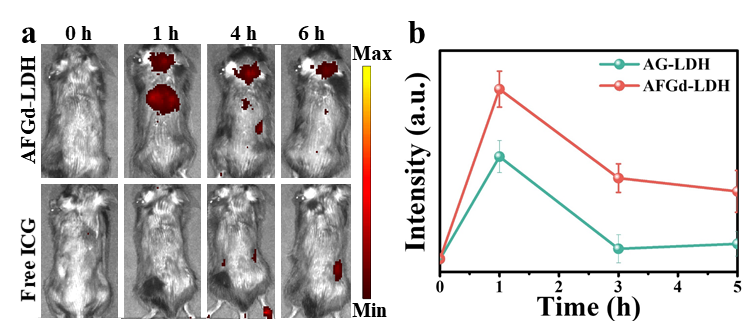
**

**Figure S14**. In vivo imaging of (a) free ICG and AFGd-LDH modified with ICG. (b) Corresponding fluorescence intensity of in vivo imaging for AGd-LDH and AFGd-LDH from Figure 4b.


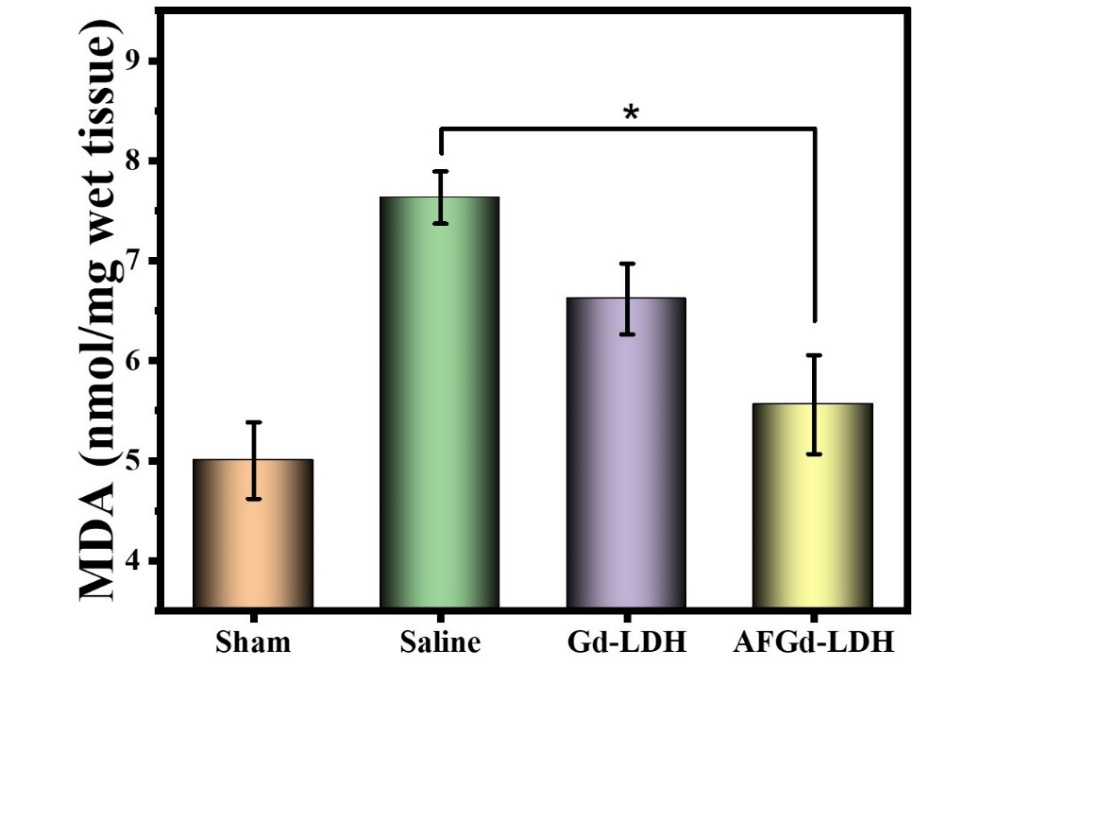


**Figure S15**. Expression levels of MDA in the brain tissue of different treatment groups (n = 3). Significant difference between treatment and control groups is indicated at *P < 0.05 levels.


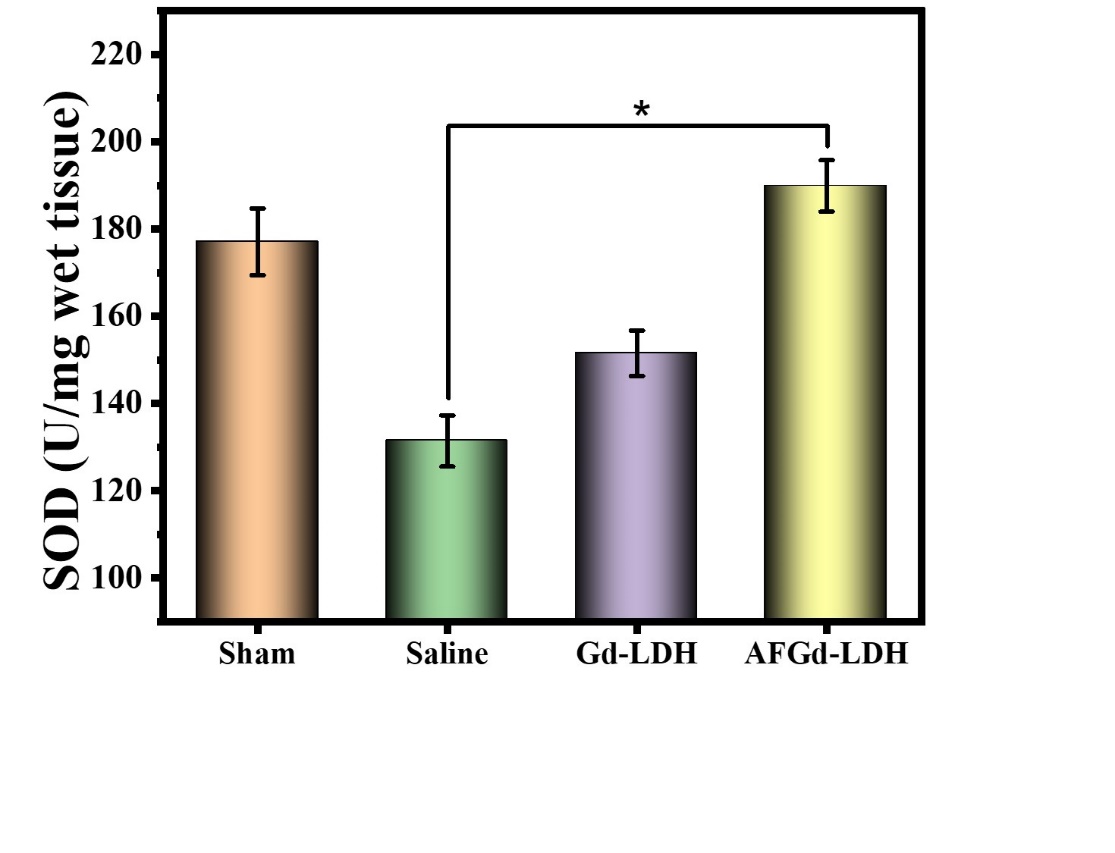


**Figure S16**. Expression levels of SOD in the brain tissue of different treatment groups (n = 3). Significant difference between treatment and control groups is indicated at *P < 0.05 levels.


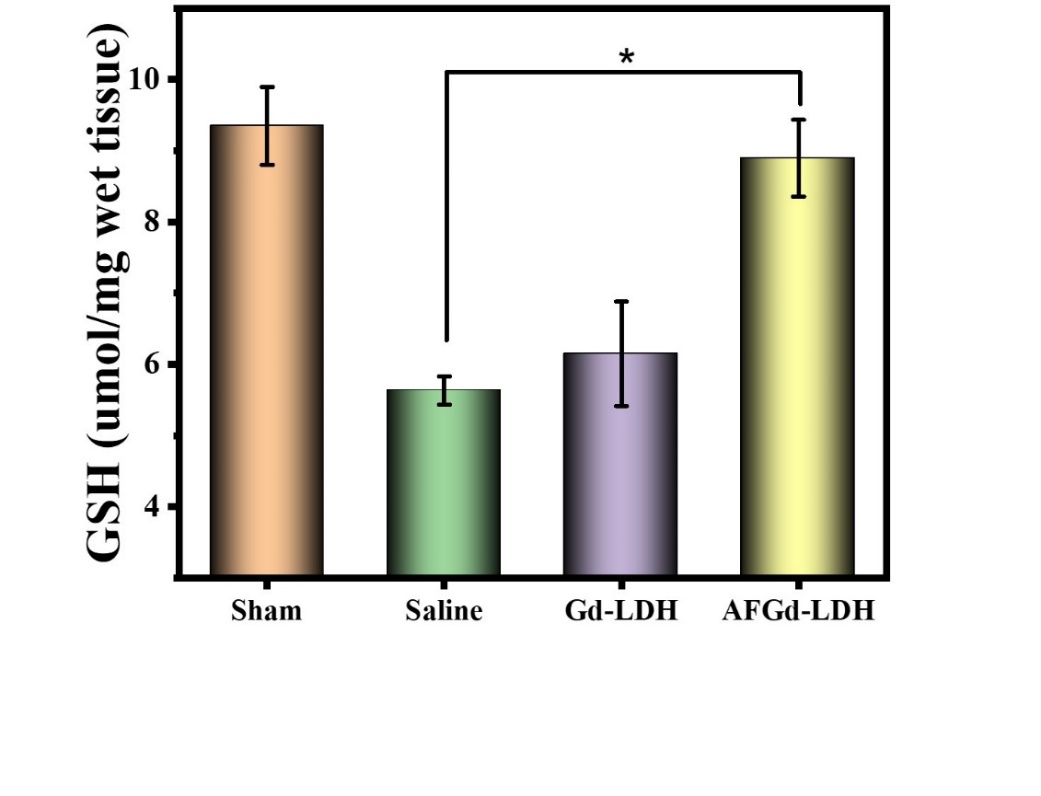


**Figure S17**. Expression levels of GSH in the brain tissue of different treatment groups (n = 3). Significant difference between treatment and control groups is indicated at *P < 0.05 levels.


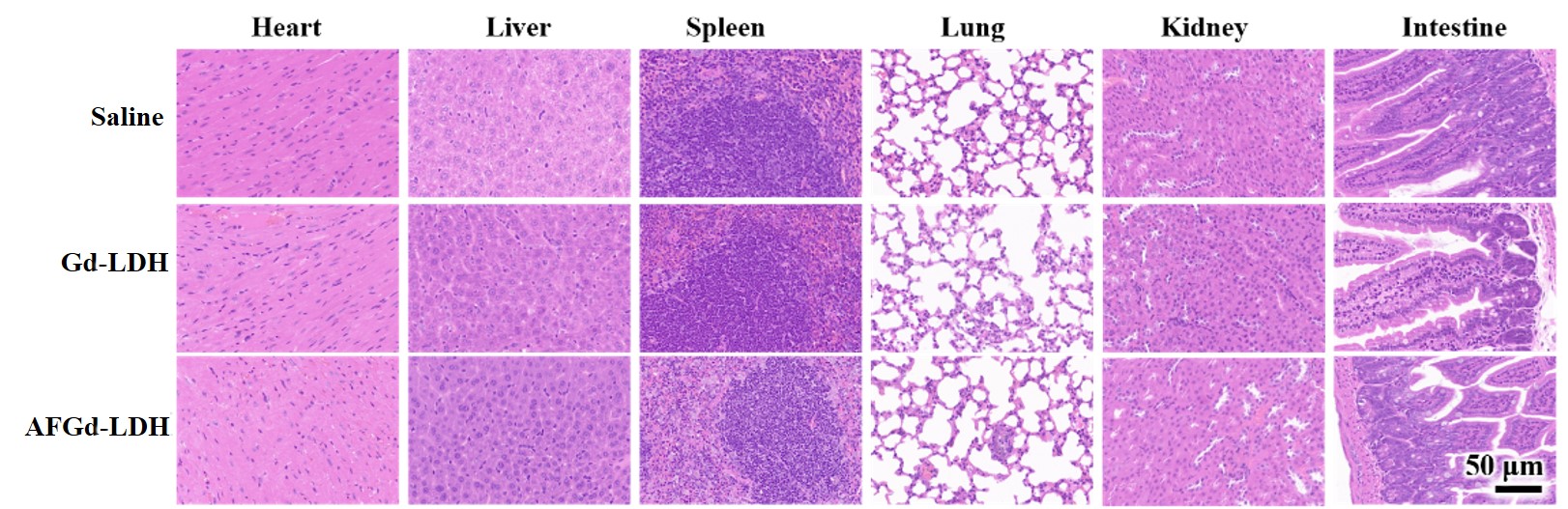


**Figure S18**. Histopathological analysis of main organs from three mice groups treated with Saline, Gd-LDH, and AFGd-LDH, respectively.
